# Comparing Open-Access Database and Traditional Intensive Care Studies Using Machine Learning: Bibliometric Analysis Study

**DOI:** 10.2196/48330

**Published:** 2024-04-17

**Authors:** Yuhe Ke, Rui Yang, Nan Liu

**Affiliations:** 1 Division of Anesthesiology and Perioperative Medicine Singapore General Hospital Singapore Singapore; 2 Centre for Quantitative Medicine Duke-NUS Medical School National University of Singapore Singapore Singapore

**Keywords:** BERTopic, critical care, eICU, machine learning, MIMIC, Medical Information Mart for Intensive Care, natural language processing

## Abstract

**Background:**

Intensive care research has predominantly relied on conventional methods like randomized controlled trials. However, the increasing popularity of open-access, free databases in the past decade has opened new avenues for research, offering fresh insights. Leveraging machine learning (ML) techniques enables the analysis of trends in a vast number of studies.

**Objective:**

This study aims to conduct a comprehensive bibliometric analysis using ML to compare trends and research topics in traditional intensive care unit (ICU) studies and those done with open-access databases (OADs).

**Methods:**

We used ML for the analysis of publications in the Web of Science database in this study. Articles were categorized into “OAD” and “traditional intensive care” (TIC) studies. OAD studies were included in the Medical Information Mart for Intensive Care (MIMIC), eICU Collaborative Research Database (eICU-CRD), Amsterdam University Medical Centers Database (AmsterdamUMCdb), High Time Resolution ICU Dataset (HiRID), and Pediatric Intensive Care database. TIC studies included all other intensive care studies. Uniform manifold approximation and projection was used to visualize the corpus distribution. The BERTopic technique was used to generate 30 topic-unique identification numbers and to categorize topics into 22 topic families.

**Results:**

A total of 227,893 records were extracted. After exclusions, 145,426 articles were identified as TIC and 1301 articles as OAD studies. TIC studies experienced exponential growth over the last 2 decades, culminating in a peak of 16,378 articles in 2021, while OAD studies demonstrated a consistent upsurge since 2018. Sepsis, ventilation-related research, and pediatric intensive care were the most frequently discussed topics. TIC studies exhibited broader coverage than OAD studies, suggesting a more extensive research scope.

**Conclusions:**

This study analyzed ICU research, providing valuable insights from a large number of publications. OAD studies complement TIC studies, focusing on predictive modeling, while TIC studies capture essential qualitative information. Integrating both approaches in a complementary manner is the future direction for ICU research. Additionally, natural language processing techniques offer a transformative alternative for literature review and bibliometric analysis.

## Introduction

The start of critical care as a medical subspecialty can be traced back to a polio epidemic during which a substantial number of patients needed prolonged mechanical ventilation [1]. Over time, the field of critical care has experienced significant growth and continual evolution. Research in this field has played a pivotal role in unraveling the complexities of numerous diseases and treatment modalities, driving substantial advancements in clinical practice over the past decades [2]. Groundbreaking studies have investigated critical areas such as sepsis, mechanical ventilation, acute lung and kidney injuries, intensive care unit (ICU) delirium, and sedation in critically ill patients [3].

These research studies have often been conducted in traditional ways such as prospective and randomized controlled trials [4], cohort and observational studies, clinical trials [5], and clinical and translational research [6]. These traditional methods have revolutionized patient care and improved outcomes significantly. For instance, the implementation of protocol-driven, goal-directed management of sepsis and appropriate fluid therapy has led to remarkable reductions in mortality rates [7,8], and these findings have been integral in developing evidence-based practice guidelines that are now the gold standard [9,10].

Despite their undeniable merits, traditional research methods in intensive care also come with several limitations [11]. Clinical trials are known for their high costs [12], stringent standardization requirements, and ethical oversight [13]. Data collection can be laborious, prone to human errors, and constrained in terms of quantity and granularity [14]. Moreover, obtaining patient consent for most randomized controlled trials in the ICU poses challenges [15], necessitating alternative consent models. These limitations have become increasingly apparent as medical complexity continues to grow exponentially [16].

The advent of electronic health records (EHRs) has heralded a new era in clinical research by facilitating the digitization of health care systems [17]. In this era of data science, a more integrated approach can be adopted, using machine learning (ML) algorithms to tackle the complexity of critical illness [18]. Open-access databases (OADs), such as the Medical Information Mart for Intensive Care (MIMIC) database [19] and the Philips eICU Collaborative Research Database (eICU-CRD) [20], have played a transformative role by enabling free data sharing.

The concept of free and open databases plays a pivotal role in promoting data sharing and advancing medical knowledge in accordance with the findable, accessible, interoperable, and reusable (FAIR) guiding principle. The FAIR principles, which emphasize that data should be findable, accessible, interoperable, and reusable, are essential for fostering a collaborative and transparent scientific research environment [21,22]. By removing barriers to access, free, and open databases allow researchers, regardless of their affiliations or resources, to contribute to and benefit from the collective pool of information. Accessibility fosters inclusivity and diversity in research, promoting a broader range of perspectives and approaches to medical challenges. This democratization of knowledge leads to a more equitable distribution of information. Researchers can now leverage these vast repositories of information for ML and artificial intelligence studies, marking a departure from traditional intensive care (TIC) research approaches.

Conducting a literature review [23] to investigate the disparities between traditional ICU research and studies based on open-access data sets holds significant importance as it provides a comprehensive understanding of the strengths and limitations of the latter. However, conventional methods of literature reviews and bibliometric analysis have their limitations, especially when dealing with large-scale literature due to computational complexity and the labor-intensive nature of manual interpretations [24-26]. To address these challenges, natural language processing (NLP) offers a promising avenue, while topic modeling techniques can be used to extract various topic themes from extensive data sets [27,28].

Built on the foundations of bidirectional encoder representations from transformers (BERT), BERTopic introduces a novel approach to topic modeling [29,30]. Unlike traditional unsupervised models like latent Dirichlet allocation, which rely on “bag-of-words” model [31], BERTopic overcomes the problem of semantic information loss, significantly enhancing the accuracy of generated topics, and providing more interpretable compositions for each topic, which greatly facilitates the classification of topics.

With the aid of BERTopic, this study aims to shed light on the disparities and commonalities between studies conducted through OADs and TIC research. By analyzing the overall trends and patterns in these 2 groups, we seek to identify knowledge gaps and explore avenues for complementary contributions between these research approaches.

## Methods

### Data

#### Data Filtering

We performed an ML-based analysis of research abstracts in the Web of Science (WoS) database to automatically categorize the research papers to conduct this literature mapping analysis. There was no limit to the year of publication of the articles. The search query consisted of the following keyword to identify all the studies that were published under the umbrella of intensive care: (“ICU” OR “intensive care”). The search terms were deliberately left to be broad to cover broad spectrums of journals in the field.

The inclusion criteria were as follows: (1) written in English, (2) articles that had keywords related to intensive care, (3) articles that had the article type of “article” or “review.” We excluded articles with incomplete data fields (eg, title, abstract, publication year, and paper citation). The articles included were then further processed to identify if they were studies using OADs. These articles were labeled as “open-access database,” while the rest of the articles extracted were labeled as “traditional intensive care.”

The search used for this study was performed on January 18, 2023, from WoS. This generated 227,893 search results, which were subsequently reselected using Python. An advanced search from PubMed was done based on the broad search terms of ICU studies used from previous Cochrane ICU literature review [32] to ensure the accuracy of the results. The numbers corroborated with a discrepancy of 4.9% (227,893 WoS keyword search vs 239,748 PubMed ICU keyword search).

#### Selection Criteria for OADs

A title search using keywords from all currently existing OADs was conducted to identify OAD studies. These include (1) MIMIC [19], (2) eICU-CRD [20], (3) Amsterdam University Medical Centers Database (AmsterdamUMCdb) [33], (4) High Time Resolution ICU Dataset (HiRID) [34], and (5) Pediatric Intensive Care database [35]. We avoided including only keywords in the search and restricted the search years by the year that the OAD was made publicly available to reduce the inadvertent inclusion of incorrect articles due to keywords. For instance, the search term for OADs published with the MIMIC database included title keyword search with the terms (“MIMIC-IV” OR “MIMIC-III” OR “MIMIC-II” OR “MIMIC Dataset” OR “medical information mart for intensive care” OR “MIMIC IV” OR “MIMIC III” OR “MIMIC II”) in studies that were published after 2003. The title keyword search for the searches and the year of cutoff for each OAD are presented in Multimedia Appendix 1.

Furthermore, to ensure the accuracy of the supervised keyword classification, a manual review of the classification by 2 critical care physicians was done for 100 articles from each category that were randomly selected. The review was done independently with the physicians labeling the extract publications into OAD and TICs. An accuracy of 99% was achieved on independent reviews, and full agreement was achieved after discussion on the discrepancy. The final results were matched with the supervised keyword classification.

We performed a bibliometric analysis by directly extracting publication details from the WoS database using Python (Python Software Foundation). The analysis involved assessing the number of articles published per year, calculating total citation counts, and identifying the top journals that published intensive care-related articles. Comprehensive results are presented in Multimedia Appendix 2.

### Data Analysis

#### Uniform Manifold Approximation and Projection

Uniform manifold approximation and projection (UMAP) is a manifold learning technique for dimension reduction, which can identify key structures in high-dimensional data space and map them to low-dimensional space to accomplish dimensionality reduction. Compared to other dimensionality reduction algorithms, such as principal component analysis [36], UMAP can retain more global features [37]. In this paper, we constructed a corpus consisting of abstract words from all studies. However, due to the massive size of the corpus, visualizing and analyzing the high-dimensional data to explore the differences in the vocabulary patterns between the OAD and TIC studies is a challenge. The UMAP package in Python, which implements the UMAP algorithm, was used to project the high-dimensional corpus to 4 dimensions. By cross plotting each dimension, we were able to investigate underlying differences in corpus distribution between OAD and TIC studies.

#### BERTopic

Topic modeling can help us explore the similarities and differences between research topics in OAD and TIC studies. Unlike conventional topic modeling models, BERTopic uses the BERT framework for embeddings, enabling a deeper understanding of semantic relationships [30]. The BERTopic model was implemented by the BERTopic package in Python and divided 146,727 studies into 30 topic IDs. We also performed latent Dirichlet allocation topic modeling through Python’s LdaModel package for comparison. Through the review of topic keywords by 2 critical care physicians, BERTopic exhibited superior accuracy and sophistication in topic identification, with enhanced interpretability and scientific rigor.

Consequently, the BERTopic model was used for the final analysis. Each of these topics was given a corresponding clinical research category. The overlapping categories were merged into topic families for easier comparisons. By using these advanced techniques, we were able to uncover hidden patterns and relationships within the literature and provide insights into the current state of intensive care research.

## Results

**Figure 1:**
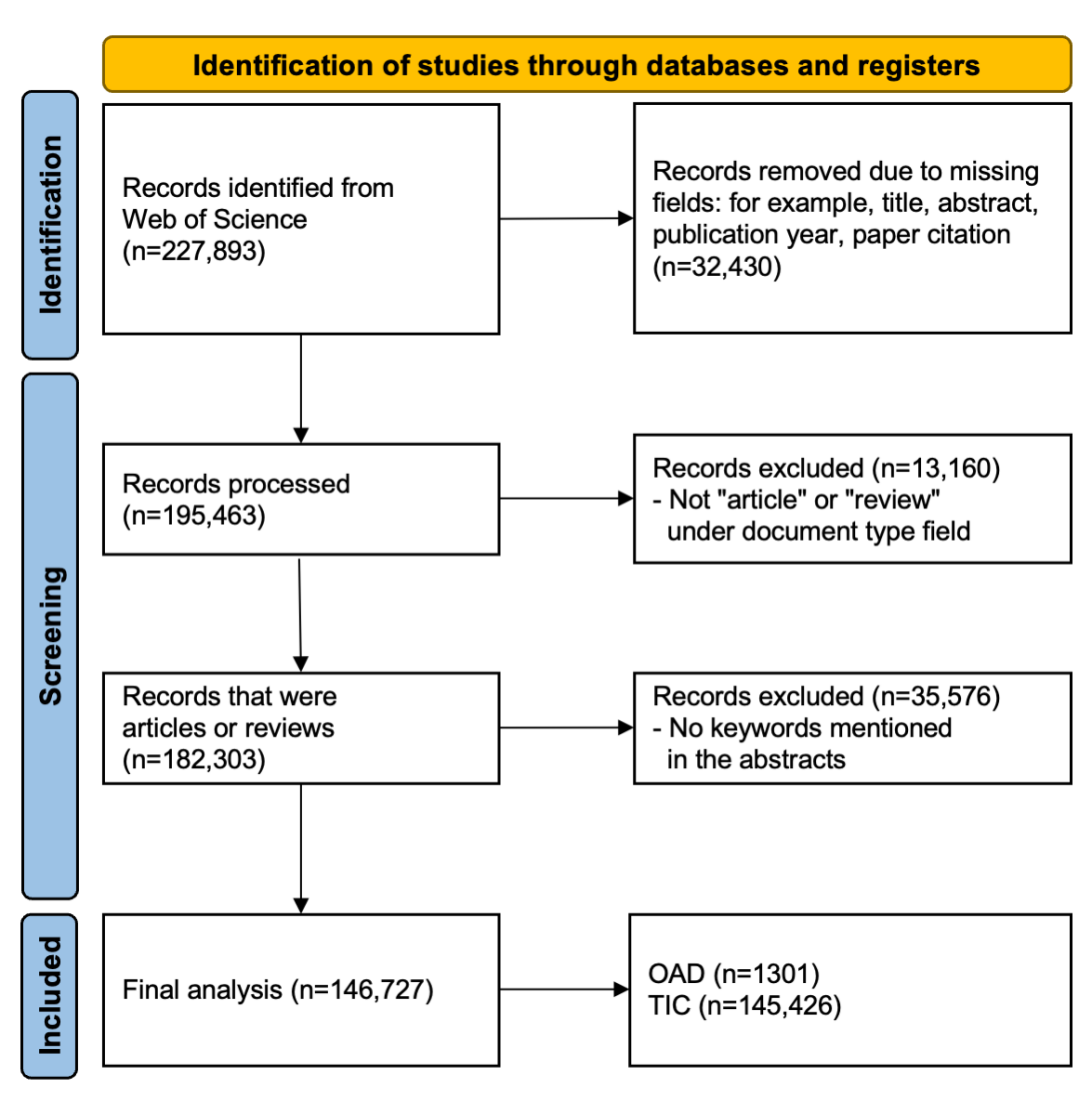
PRISMA (Preferred Reporting Items for Systematic Review and Meta-Analysis) diagram of the study. The final studies were divided into open-access database (OAD) and traditional intensive care (TIC) studies.

A total of 227,893 records were identified from the WoS database on January 18, 2023, of which 195,463 full records were subsequently processed. Records were excluded if they are not “article” or “review” or if they do not contain keywords related to intensive care. After exclusions, 145,426 articles were identified as TIC studies and 1301 articles were categorized as OAD (Figure 1).

**Figure 2:**
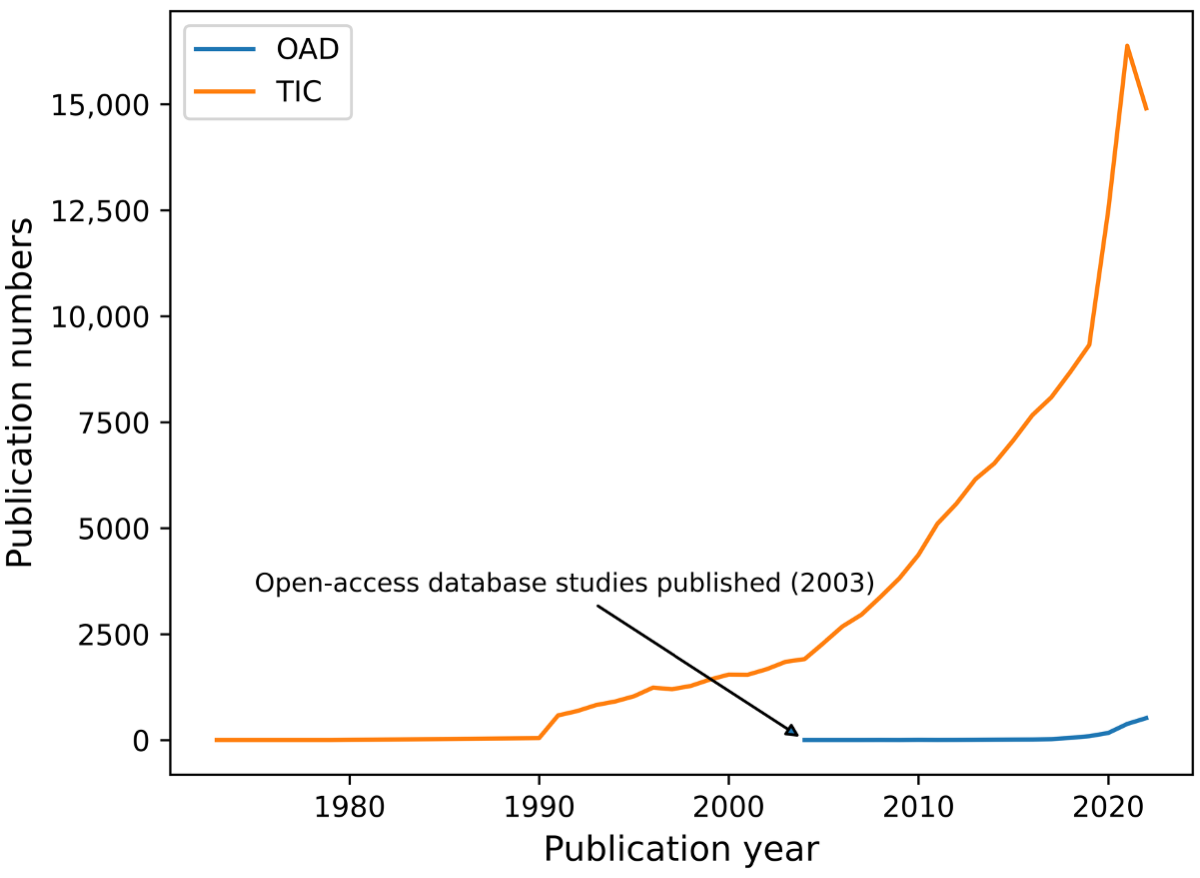
Number of publications of open-access database (OAD) and traditional intensive care (TIC) studies by year. The first study in the OAD category started in the year 2003.

We examined the number of articles published per year to analyze the trends in TIC and OAD studies (Figure 2). Over the past 2 decades, TIC studies have experienced exponential growth, culminating in a peak of 16,378 articles in 2021. A subsequent decline in the number of publications occurred in 2022, likely attributable to delayed indexing within the WoS database and a reduction in COVID-19–related studies as the pandemic stabilized [38]. In contrast, the first OAD study emerged in 2003, with its popularity experiencing a consistent upsurge since 2018. Nonetheless, the number of OAD publications remains markedly lower in comparison to TIC publications.

The OAD studies were published most frequently in new open-access journals such as *Frontiers in Medicine*, *Frontiers in Cardiovascular Medicine*, and *Scientific Reports* while the TIC studies were published most frequently in established journals like *Critical Care Medicine*, *Intensive Care Medicine*, and *Critical Care* (Multimedia Appendix 2). Further analysis of keywords from the abstracts showed 2.4% (3492/145,426) TIC studies were meta-analyses or systematic reviews, while only 0.08% (1/1301) OAD study was in this category. There were 5.61% (73/1301) OAD studies, and 7.43% (10,799/145,426) TIC studies that had the keyword of “cost.” Examples of the data fields that are available within OADs such as MIMIC and eICU-CRD are listed in Textbox 1. Some information fields such as end-of-life goals and values and health care provider psychology are not available within the current EHRs extracted for OADs.

**Examples of information available in the current open-access databases (OADs) and examples of information not available in OADs.**

**Examples of information that is available in current OADs**

- Patient information: demographics and social set-up
- Hospital context: admission time and discharge time, intensive care unit (ICU) and hospital admissions, and pre-ICU admission
- Diagnosis: physician-curated ICU diagnosis and data-driven phenotypes
- Intervention: medications, procedures, and organ support
- Diagnostics: blood test, microbiology, and scans
- Clinical texts: clinical notes and diagnostic reports
- Physiological monitoring: basic monitoring and waveforms

**Examples of information that is not readily available in current OADs**

- Patient information: family set up and visiting, financial information, and special populations
- Hospital context: post-ICU discharge details, delayed admission or discharge, and health personnel psychology
- Diagnosis: pre-ICU history and diagnosis requiring clinical symptoms
- Intervention: indications for interventions, complications, and intraoperative and postoperative
- Diagnostics: pathology photographs, imaging, and molecular or genetic studies
- Clinical texts: patient narratives, end-of-life goals and patient value, and health personnel behavior
- Physiological monitoring: advanced monitoring

**Figure 3:**
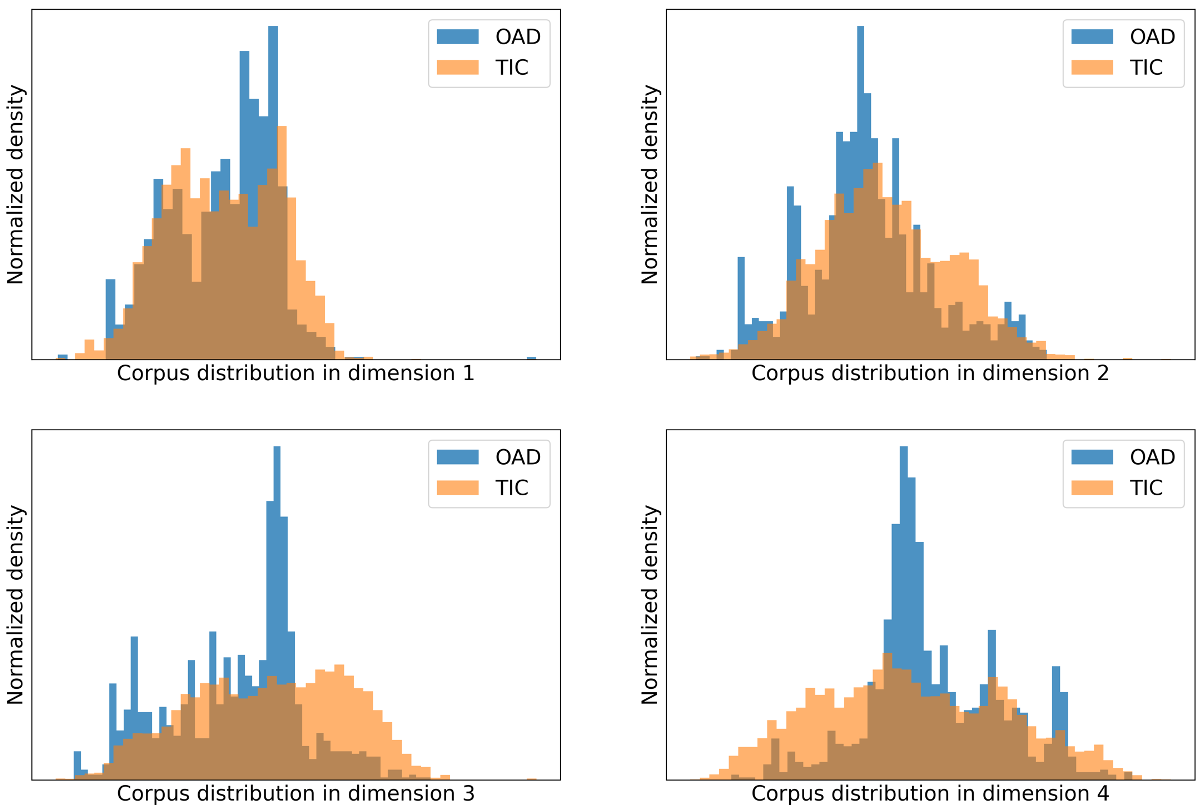
Corpus distribution of open-access database and traditional intensive care studies along dimensions 1-4. OAD: open-access database; TIC: traditional intensive care.

The UMAP algorithm was used to project the high-dimension corpus to 4 dimensions and allowed exploration of the vocabulary patterns between the OAD and TIC studies (Figure 3). The projection values are represented by the x-axis, while the densities are represented by the y-axis. The presence of considerable overlap between TIC studies and OAD studies suggests that they share a substantial number of common terminologies, which may correspond to similar research topics. Nonetheless, TIC studies exhibit a more extensive coverage than OAD studies, which may stem from broader research scope and extended research duration.

**Figure 4:**
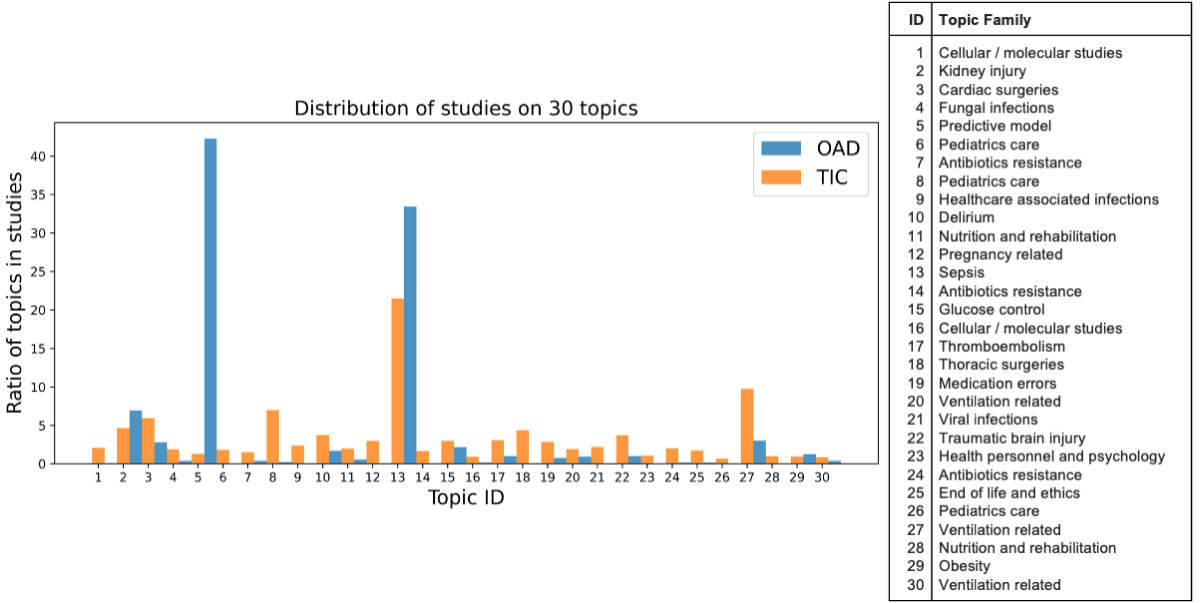
The ratio of open-access database and traditional intensive care studies within each topic was identified by BERTopic.

Subsequently, the BERTopic model was then used to generate 30 topic IDs (Figure 4). The internal commonalities of each topic ID were reviewed by critical care physicians and assigned a specific subtopic in intensive care research. The model was able to automatically classify the topics with high interpretability and the topic components were interpreted with ease. For instance, components in topic ID 5 consist of, in decreasing order of weightage: “learning,” “model,” “machine,” “machine learning,” “models,” “data,” “prediction,” and “performance.” This topic was consequently labeled “predictive model” (topic ID 5 in Multimedia Appendix 3).

The overall topic distribution in TIC studies was more uniform, while the OAD studies tended to be concentrated on several topics including topic ID 2 (kidney injury), 5 (predictive model), and 13 (sepsis). Some topics that were missing in OAD studies included 6 (pediatrics care), 21 (viral infections), 23 (health personnel and psychology), and 28 (nutrition and rehabilitation).

The similarity matrix shows that there was little overlap between the topics (Multimedia Appendix 4). To facilitate the interpretability of the categories, the overlapping topic IDs were merged to form the final 22 topic families (Multimedia Appendix 3).

**Figure 5:**
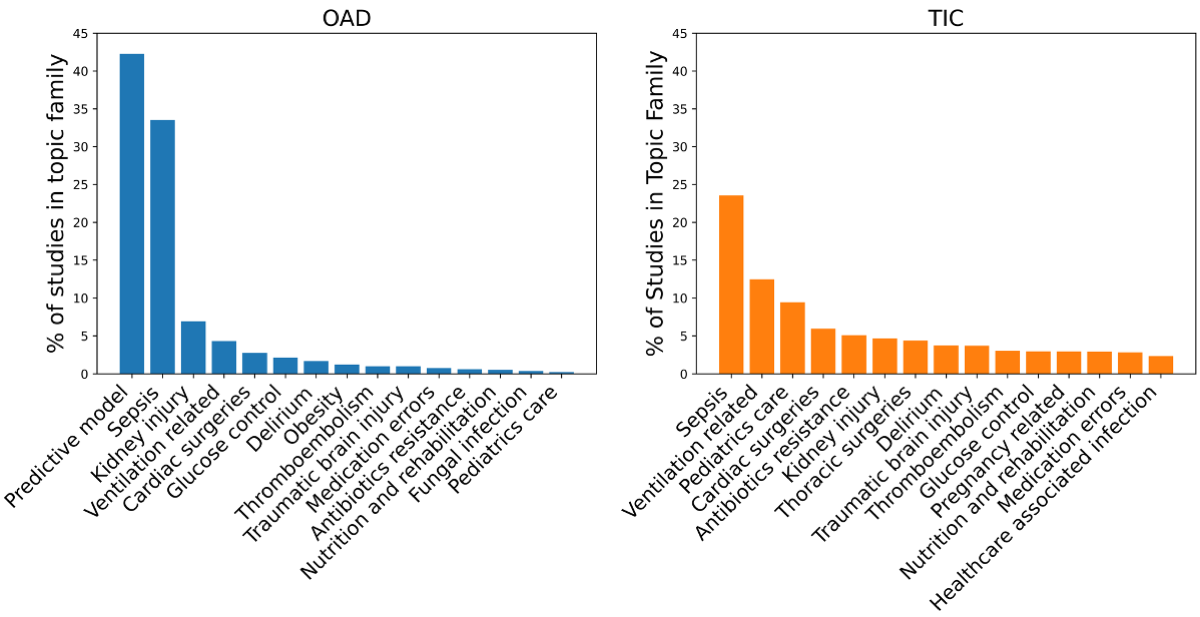
The top 15 topic families represented in open-access database and traditional intensive care studies.

Topics such as “healthcare associated infection,” “thoracic surgeries,” and “pregnancy related” research were among the more frequently discussed 15 topics in TIC studies but have limited publications in OAD studies. The topics of “predictive model,” “obesity,” and “fungal infections” were popular in OAD studies but not the TIC studies. Overall, the topic distributions of the TIC studies were distributed more evenly with the topic family of sepsis accounting for a quarter of the studies, while publications in the OAD studies were heavily skewed toward the predictive model (>40%) and sepsis (>30%; Figure 5).

## Discussion

### Principal Results

This study conducted a comprehensive review and bibliometric analysis of OAD and TIC studies. NLP was used to facilitate this large-scale literature review. Studies using OADs mainly concentrated on a few topics, such as predictive modeling, while TIC studies covered a wider range of topics with a more balanced distribution.

### Advantages of OAD Studies

OAD studies offer several advantages that have contributed to their increasing popularity in intensive care research. The granularity of data and easy access to large-cohort databases, such as MIMIC [39], has enabled researchers to perform predictive modeling and conduct various secondary analyses efficiently [40,41]. This accessibility has provided valuable opportunities for exploring specific aspects of patient care, evident in studies investigating phenomena like “weekend effects” and circadian rhythms in ICU patients before discharge [42-46]. The vast amount of longitudinal and time series data available in OADs has also facilitated the implementation of complex ML and deep learning methods [47].

### Limitations of OAD Studies

However, it is crucial to acknowledge the retrospective nature of OAD data, which inherently limits the assessment of confounding factors and the ability to draw strong causal conclusions. The observational design of OAD studies may result in lower-quality evidence according to the GRADE (Grading of Recommendations, Assessment, Development, and Evaluations) framework [48,49], and thus, the research from OAD studies has yet to be fully integrated into existing evidence-based guidelines, as exemplified by the omission of OAD studies in the 2021 sepsis guidelines [50]. Nevertheless, OADs remain a valuable resource for supplementing and complementing TIC studies, providing unique insights and enhanced predictive scores for intensive care settings.

Furthermore, approximately 50% of the studies using OADs published focused on predictive modeling. The increased usage of ML methods in predictive modeling has not been without critique. Some medical prediction problems inherently possess linear characteristics, and the selection of features may predominantly focus on already known strong predictors, leading to limited improvements in prediction accuracy with ML [51]. Additionally, interstudy heterogeneity poses a challenge in comparing results obtained from different ML models applied to the same data sets [52]. The ethical implications of relying solely on ML models to make high-risk health care decisions instead of involving clinical expertise are also relevant considerations [51,53].

While OADs provide comprehensive patient data, there are certain limitations in their ability to capture specific information essential for certain critical care research areas. Notably, data fields related to qualitative aspects such as ethics and end-of-life care [54,55], and health care personnel psychology [56] may be challenging, if not impossible, to obtain through OADs generated from EHRs. Consequently, TIC studies have played a pivotal role in addressing these limitations by capturing critical information that is integral to understanding ethical considerations, patient experiences, and health care provider psychology in intensive care [57,58].

### Synergy Between OAD and TIC Studies

The synergy between OAD and TIC studies is a promising approach to enhance the comprehensiveness and robustness of intensive care research. OADs, with their large cohort sizes, can serve as external validation cohorts for ML models developed from TIC studies, potentially reducing the sample sizes required for prospective research. Furthermore, OAD studies can corroborate the results of TIC studies, benefiting from larger sample sizes and real-world data, thus providing more practical insights for implementation in intensive care settings [43]. The integration of OAD and TIC studies presents an opportunity to bridge the gaps in data availability and research methodologies, ultimately enriching the understanding and practice of critical care medicine.

### Potential Impact of NLP

The usage of large language models such as BERTopic has proven to be a valuable tool for large-scale literature review and topic extraction [58]. This approach has enabled accurate, reliable, and granular topic generation, offering clinicians a more effective means of interpreting data compared to traditional bag-of-words models [59]. The potential of NLP to analyze scientific articles and identify trends and knowledge gaps holds promise for shaping the future of research in critical care medicine. As the volume of publications in critical care continues to grow and large language modeling continues to advance in health care [60], AI technology will be crucial in efficiently identifying and predicting emerging trends.

### Future Directions

Future research in the field of critical care can explore novel applications of ML beyond predictive modeling. For instance, using ML to study patterns in how papers are cited, shared, and discussed on the web could help predict their potential impact on the scientific community. This analysis can aid in identifying highly influential papers and understanding the factors that contribute to their recognition. Additionally, investigations into methods for enhancing the interpretability and transparency of ML algorithms in critical care research would further facilitate the ethical and responsible use of AI technologies.

### Strengths and Limitations

The study’s application of NLP-driven in analyzing scientific articles and identifying trends highlights the potential impact of AI technologies in streamlining literature reviews and identifying emerging trends more efficiently.

Another notable strength of this study is the usage of the WoS database, the world’s oldest and most extensively used repository of research publications and citations, encompassing approximately 34,000 journals [61]. The comprehensiveness of this database provides a robust representation of the literature in the field of intensive care research. Nevertheless, it is essential to acknowledge that some articles published in nonindexed journals might not have been captured, and future studies could benefit from considering additional databases to supplement our findings.

One other limitation lies in the classification of OAD and TIC studies, which may be subject to variations in the interpretation of keywords. However, we optimized the keyword combinations during the search process in the WoS database and implemented Python filtering techniques, resulting in a relatively high level of accuracy in our classifications. The number of studies was further corroborated with a manual search on PubMed and a review of the classifications of the studies was done by critical care physicians.

Although there were no specific language restrictions, the nature of the search term being in English inadvertently excluded valuable contributions from non-English research. This may potentially limit the generalizability of our findings to a broader international audience. In future investigations, the inclusion of articles from various languages could offer a more comprehensive and diverse perspective on intensive care research.

### Conclusions

This study has provided valuable insights into the expanding landscape of intensive care research through a comprehensive bibliometric analysis of a large number of publications by leveraging NLP technologies. While OAD studies have demonstrated significant promise, it is essential to view them as a complementary approach rather than a replacement for TIC studies. The unique strength of TIC studies lies in their ability to capture crucial qualitative information, which is essential for comprehensive and ethical decision-making. The integration of both OAD and TIC studies offers a synergistic approach to enriching our understanding of critical care medicine and advancing patient care outcomes. As NLP technology continues to advance, it holds the potential to offer a feasible and transformative alternative for literature review and bibliometric analysis.

## Appendix

Multimedia Appendix 1 Search terms for open-access database (OAD) studies with the cutoff by the years of publications.

Multimedia Appendix 2 Top 20 journals ranked by total citation in which the open-access database and traditional intensive care studies were published. The average citation per article was obtained with the total citation/total number of articles. The citation counts were obtained from Web of Science.

Multimedia Appendix 3 Topic ID and topic family and the components and weightage in each of the categories.

Multimedia Appendix 4 Similarity matrix of 30 topics.

## Abbreviations

AmsterdamUMCdb: Amsterdam University Medical Centers Database
BERT: bidirectional encoder representations from transformers
EHR: electronic health record
eICU-CRD: eICU Collaborative Research Database
FAIR: findable, accessible, interoperable, and reusable
GRADE: Grading of Recommendations, Assessment, Development, and Evaluations
HiRID: High Time Resolution ICU Dataset
ICU: intensive care unit
MIMIC: Medical Information Mart for Intensive Care
ML: machine learning
NLP: natural language processing
OAD: open-access database
TIC: traditional intensive care
UMAP: uniform manifold approximation and projection
WoS: Web of Science

## Data Availability

The data sets generated during and/or analyzed during this study are available from the corresponding author on reasonable request. The complete set of code used in this study is readily available for download on GitHub [62].
