# Supplementary material for: Comparing Open-Access Database and Traditional Intensive Care Studies Using Machine Learning: Bibliometric Analysis Study
Source: J Med Internet Res. 2024 Apr 17;26:e48330. doi: 10.2196/48330 (PMC11063894; doi:10.2196/48330)
Supplement: Multimedia Appendix 1 [file jmir_v26i1e48330_app1.docx]

**Multimedia Appendix 1:** Search terms for Open-Access database (OAD) studies with the cut-off by the years of publications.

| Search terms | Year of qualification |
| --- | --- |
| Open-Access Database (OAD) studies | |
| Title keyword search for “MIMIC-IV” or “MIMIC-III” or “MIMIC-II” or “MIMIC Dataset” or “medical information mart for intensive care” or “MIMIC IV” or “MIMIC III” or “MIMIC II” | 2003 |
| Title keyword search for “eICU-CRD” or “eICU” or “eICU Collaborative Research Database” | 2018 |
| Title keyword search for “AmsterdamUMCdb” or “Amsterdam University Medical Centers Database” | 2021 |
| Title keyword search for “HiRID” or “High time resolution ICU dataset” | 2020 |
| Title keyword search for “Pediatric Intensive Care database” or “Pediatric Intensive Care Unit database” | 2020 |
