# Supplementary material for: Comparing Open-Access Database and Traditional Intensive Care Studies Using Machine Learning: Bibliometric Analysis Study
Source: J Med Internet Res. 2024 Apr 17;26:e48330. doi: 10.2196/48330 (PMC11063894; doi:10.2196/48330)
Supplement: Multimedia Appendix 2 [file jmir_v26i1e48330_app2.docx]

**Multimedia Appendix 2:** Top 20 journals ranked by total citation (TC) where the OAD and TIC studies were published in. The Average citation per article (AC) was obtained with the total citation/total number of articles. The citation counts were obtained from WoS.

| **Open-Access Database (OAD)(N=1,301)** | | | | **Traditional Intensive Care (TIC)(N=145,426)** | | | |
| --- | --- | --- | --- | --- | --- | --- | --- |
|  | **No. of Article** | **TC** | **AC** | **Journal** | **No. of Article** | **TC** | **AC** |
| **Journal** |  |  |  |  |  |  |  |
| Frontiers In Medicine | 61 | 150 | 2.46 | Critical Care Medicine | 4,958 | 372,139 | 75.06 |
| Frontiers In Cardiovascular Medicine | 45 | 63 | 1.4 | Intensive Care Medicine | 3,166 | 196,056 | 61.93 |
| Scientific Reports | 43 | 973 | 22.63 | Critical Care | 2,444 | 120,649 | 49.37 |
| International Journal Of General Medicine | 38 | 120 | 3.16 | Journal Of Critical Care | 1,927 | 40,428 | 20.98 |
| Journal Of Biomedical Informatics | 35 | 658 | 18.8 | Plos One | 1,777 | 32,748 | 18.43 |
| Plos One | 31 | 375 | 12.1 | Pediatric Critical Care Medicine | 1,332 | 32,636 | 24.5 |
| Ieee Journal Of Biomedical And Health Informatics | 24 | 210 | 8.75 | Chest | 1,288 | 101,947 | 79.15 |
| Ieee Access | 23 | 223 | 9.7 | Indian Journal Of Critical Care Medicine | 794 | 5,316 | 6.7 |
| Annals Of Translational Medicine | 23 | 82 | 3.57 | Bmj Open | 780 | 7,607 | 9.75 |
| Bmc Medical Informatics And Decision Making | 23 | 175 | 7.61 | Infection Control And Hospital Epidemiology | 743 | 28,838 | 38.81 |
| Journal Of The American Medical Informatics Association | 22 | 596 | 27.09 | Medicine | 741 | 6,852 | 9.25 |
| Bmj Open | 19 | 112 | 5.89 | Annals Of Intensive Care | 712 | 15,623 | 21.94 |
| Computers In Biology And Medicine | 18 | 276 | 15.33 | Journal Of Hospital Infection | 705 | 22,029 | 31.25 |
| Critical Care | 17 | 354 | 20.82 | Journal Of Cardiothoracic And Vascular Anesthesia | 699 | 12,066 | 17.26 |
| Bmc Cardiovascular Disorders | 17 | 18 | 1.06 | American Journal Of Infection Control | 670 | 17,754 | 26.5 |
| Jmir Medical Informatics | 16 | 86 | 5.38 | Acta Anaesthesiologica Scandinavica | 655 | 12,046 | 18.39 |
| Frontiers In Pharmacology | 15 | 10 | 0.67 | Journal Of Clinical Medicine | 654 | 3,685 | 5.63 |
| Frontiers In Neurology | 14 | 11 | 0.79 | Scientific Reports | 648 | 5,684 | 8.77 |
| Computer Methods And Programs In Biomedicine | 13 | 110 | 8.46 | Journal Of Intensive Care Medicine | 640 | 5,672 | 8.86 |
| Disease Markers | 13 | 51 | 3.92 | Journal Of Perinatology | 613 | 12,177 | 19.86 |
