# Supplementary material for: Comparing Open-Access Database and Traditional Intensive Care Studies Using Machine Learning: Bibliometric Analysis Study
Source: J Med Internet Res. 2024 Apr 17;26:e48330. doi: 10.2196/48330 (PMC11063894; doi:10.2196/48330)
Supplement: Multimedia Appendix 3 [file jmir_v26i1e48330_app3.docx]

**Multimedia Appendix 3:** Topic ID and Topic Family and the components and weightage in each of the categories.

| Topic ID | Components within the topic | Topic Family |
| --- | --- | --- |
| 1 | [('sepsis', 0.27332579659105266),  ('il', 0.2349331769309052),  ('pct', 0.20688752428615256),  ('septic', 0.20365692356015097),  ('levels', 0.19867140754643542),  ('inflammatory', 0.19382192805883686),  ('expression', 0.18262226882708676),  ('crp', 0.16998930870275195),  ('cells', 0.16756787003509055),  ('plasma', 0.16651943559026977)], | Cellular / Molecular Studies |
| 2 | [('aki', 0.36854096036896355),  ('kidney', 0.26803538053596204),  ('renal', 0.2492702459064692),  ('kidney injury', 0.2290889921121861),  ('acute kidney', 0.22591782716599376),  ('acute kidney injury', 0.22391387249500308),  ('rrt', 0.21009784481870297),  ('dialysis', 0.1989376507539242),  ('creatinine', 0.1950728986720971),  ('crrt', 0.19442507897207584)], | Kidney Injury |
| 3 | [('valve', 0.21388880831731774),  ('surgery', 0.2110235495321011),  ('bypass', 0.2102351250008794),  ('coronary', 0.20801064687363396),  ('cabg', 0.20256626306305658),  ('artery', 0.19920164836051968),  ('postoperative', 0.19627109385515665),  ('aortic', 0.19604567143976592),  ('coronary artery', 0.19173844557099723),  ('cardiac', 0.1889060492354533)], | Cardiac Surgeries |
| 4 | [('candida', 0.3751145459677185),  ('antifungal', 0.29324340177333763),  ('candidemia', 0.2786999995318872),  ('albicans', 0.27275352451739526),  ('fungal', 0.2721322157200816),  ('fluconazole', 0.2511421539666509),  ('species', 0.23636576208033636),  ('candidiasis', 0.22704706750105763),  ('invasive', 0.2143637306475624),  ('parapsilosis', 0.20314387246326104)], | Fungal Infections |
| 5 | [('learning', 0.24762436454254644),  ('model', 0.23919783724967467),  ('machine', 0.2277647996671158),  ('machine learning', 0.2208741614743188),  ('models', 0.21917924275078182),  ('data', 0.2134730718570196),  ('prediction', 0.2083190692676851),  ('performance', 0.18523466000322705),  ('proposed', 0.18311637200200215),  ('based', 0.17508568370026323)], | Predictive Model |
| 6 | [('parents', 0.3077109988220944),  ('mothers', 0.2966183807553611),  ('nicu', 0.2598615277023844),  ('infant', 0.23440491157590507),  ('music', 0.23056263022156764),  ('infants', 0.21596297671035686),  ('family', 0.20972225662627964),  ('parental', 0.20458603442124007),  ('fathers', 0.20196773686596756),  ('care', 0.19821408847464242)], | Pediatrics Care |
| 7 | [('esbl', 0.29828344298195997),  ('isolates', 0.2922588725520251),  ('pneumoniae', 0.27526410276138424),  ('producing', 0.25119692574393826),  ('vre', 0.25032120868744184),  ('resistant', 0.24779732824850215),  ('resistance', 0.24057439901773933),  ('strains', 0.23453907695146253),  ('bla', 0.2341540225889985),  ('klebsiella', 0.227157756121758)], | Antibiotics Resistance |
| 8 | [('rop', 0.31339610257716577),  ('infants', 0.29081785419401157),  ('neonatal', 0.2567962472088666),  ('birth', 0.23741536525493664),  ('preterm', 0.21811383605954415),  ('weeks', 0.21220840274413547),  ('nicu', 0.20221507009073564),  ('neonatal intensive', 0.19861851788454488),  ('birth weight', 0.19860313254530754),  ('neonatal intensive care', 0.19851454030847868)], | Pediatrics Care |
| 9 | [('catheter', 0.34689212056375235),  ('catheters', 0.2605411881808324),  ('central', 0.255065304755493),  ('clabsi', 0.24107315819609532),  ('infection', 0.23254251375717974),  ('infections', 0.23190278962803276),  ('cvc', 0.22406566430488953),  ('bloodstream', 0.20890731301220583),  ('venous', 0.2060217644803757),  ('central venous', 0.20493363812340082)], | Healthcare Associated Infections |
| 10 | [('delirium', 0.5154588148984678),  ('cognitive', 0.2243747689705874),  ('cam', 0.21847014369246343),  ('cam icu', 0.20608901382412773),  ('postoperative delirium', 0.20389931843713457),  ('confusion assessment', 0.19746130119400274),  ('confusion assessment method', 0.19652113398650564),  ('assessment method', 0.19635137703237093),  ('pod', 0.1933820336941744),  ('confusion', 0.1923434101105709)], | Delirium |
| 11 | [('nutrition', 0.330371538389155),  ('nutritional', 0.2746524243695784),  ('enteral', 0.2724420887669528),  ('energy', 0.23119431820502706),  ('feeding', 0.23047240236226227),  ('enteral nutrition', 0.22244133664534557),  ('en', 0.21577335971994369),  ('intake', 0.20162364162764768),  ('malnutrition', 0.1944767915253007),  ('protein', 0.19347007646511274)], | Nutrition and Rehabilitation |
| 12 | [('women', 0.301399240757201),  ('delivery', 0.2526601882181523),  ('maternal', 0.2503067670663095),  ('pregnancy', 0.24804705850274059),  ('neonatal', 0.24082609966160043),  ('cesarean', 0.22655482681236563),  ('gestational', 0.22475623910723805),  ('pregnancies', 0.22389821011986893),  ('fetal', 0.22332508626261072),  ('perinatal', 0.21386761581786307)], | Pregnancy Related |
| 13 | [('sepsis', 0.3123750798571854),  ('qsofa', 0.21595197079345102),  ('severe sepsis', 0.2133011255394017),  ('mortality', 0.20095798517246147),  ('septic', 0.18908905733985276),  ('septic shock', 0.1829766005399198),  ('score', 0.17826212324989094),  ('shock', 0.1779489095080336),  ('hospital', 0.16619857991376882),  ('patients', 0.1644323632051158)], | Sepsis |
| 14 | [('baumannii', 0.40588714150046956),  ('isolates', 0.3245889362944371),  ('acinetobacter', 0.308954311291679),  ('aeruginosa', 0.27060997647380397),  ('resistance', 0.25885574598985206),  ('strains', 0.2583275466250913),  ('resistant', 0.2553449291766748),  ('acinetobacter baumannii', 0.25342889158867504),  ('oxa', 0.23583838379501437),  ('crab', 0.21995912556992822)], | Antibiotics Resistance |
| 15 | [('glucose', 0.4461316568965774),  ('insulin', 0.37228399269849577),  ('blood glucose', 0.32487626417375104),  ('hyperglycemia', 0.29735149084349966),  ('mg dl', 0.2818241049334382),  ('glycemic', 0.2775047690390568),  ('hypoglycemia', 0.27361029132178355),  ('dl', 0.2713467853200931),  ('bg', 0.23942916775974407),  ('glycemic control', 0.23494065660163552)], | Glucose Control |
| 16 | [('cartilage', 0.2526798553604946),  ('collagen', 0.22134806261782417),  ('ii', 0.20998865527280222),  ('cells', 0.20653654341999123),  ('ca2', 0.19550316392123837),  ('complexes', 0.19317738867529374),  ('metal', 0.189841802653695),  ('chondrocytes', 0.1896976166185451),  ('mimic', 0.18688959860617935),  ('cell', 0.1852917711758701)], | Cellular / Molecular Studies |
| 17 | [('vte', 0.33269756623068625),  ('covid', 0.28849663356259003),  ('covid 19', 0.2867096155899883),  ('19', 0.273532931388593),  ('dvt', 0.26690666750580344),  ('thrombosis', 0.256100233450463),  ('thromboembolism', 0.22826484264521532),  ('venous', 0.21890147501450632),  ('venous thromboembolism', 0.2142113867971442),  ('thromboprophylaxis', 0.21297735095553197)], | Thromboembolism |
| 18 | [('resection', 0.2681692364209983),  ('esophagectomy', 0.22754780465725122),  ('postoperative', 0.21802996064882885),  ('cancer', 0.20572583103821854),  ('laparoscopic', 0.20234694233593287),  ('surgery', 0.2016667432621023),  ('complications', 0.2002871974734891),  ('vats', 0.19595251063699398),  ('esophageal', 0.1914686070271431),  ('mie', 0.18677090359957094)], | Thoracic Surgeries |
| 19 | [('medication', 0.3537710342887063),  ('errors', 0.3517664960479983),  ('drug', 0.3376030645727846),  ('medication errors', 0.2608046032446885),  ('drugs', 0.23946456557548185),  ('pharmacist', 0.2231172198161811),  ('error', 0.2133295731022147),  ('medications', 0.2102962737554977),  ('pharmacists', 0.2070231454113488),  ('adverse drug', 0.20601508300491111)], | Medication Errors |
| 20 | [('niv', 0.3773031912155193),  ('copd', 0.3214925815321429),  ('hfnc', 0.2794037778114032),  ('failure', 0.23938927179708316),  ('respiratory failure', 0.23711423362621034),  ('respiratory', 0.2304368168407322),  ('ventilation', 0.21894186006004238),  ('noninvasive', 0.2106989777880244),  ('exacerbation', 0.20889582031488574),  ('intubation', 0.20769718304948825)], | Ventilation Related |
| 21 | [('influenza', 0.5158352377575757),  ('h1n1', 0.385500269578629),  ('influenza h1n1', 0.27298797712344364),  ('2009', 0.2644389295398752),  ('pandemic', 0.22271858342616374),  ('virus', 0.21880745619477035),  ('oseltamivir', 0.20346570605961128),  ('vaccination', 0.2006128755347512),  ('h1n1 influenza', 0.1943350127990913),  ('antiviral', 0.1908148071570294)], | Viral Infections |
| 22 | [('tbi', 0.3362258131055145),  ('icp', 0.28611068371843607),  ('injury', 0.2727932976847282),  ('brain', 0.2530286083464144),  ('brain injury', 0.23943894753075481),  ('traumatic brain', 0.23718643918611215),  ('traumatic brain injury', 0.2316906321618808),  ('intracranial', 0.22800336964985202),  ('injuries', 0.22615987848410102),  ('traumatic', 0.22468824683961797)], | Traumatic Brain Injury |
| 23 | [('nurses', 0.3483258347018276),  ('burnout', 0.33960388256490803),  ('moral', 0.29996553277198296),  ('moral distress', 0.2926145057357521),  ('work', 0.2521062095066761),  ('safety', 0.24944492943113),  ('working', 0.2394157065012202),  ('job', 0.23221917481201917),  ('distress', 0.21794057622522842),  ('stress', 0.2175821275308836)], | Health Personnel and Psychology |
| 24 | [('mrsa', 0.5702390110783249),  ('aureus', 0.35274605121151137),  ('methicillin', 0.3167304912931998),  ('staphylococcus', 0.2888558710060983),  ('methicillin resistant', 0.2841413247989383),  ('isolates', 0.27553547260326433),  ('staphylococcus aureus', 0.26871717462080835),  ('aureus mrsa', 0.26365615789640834),  ('resistant', 0.25734100111719554),  ('resistant staphylococcus', 0.256692339103793)], | Antibiotics Resistance |
| 25 | [('palliative', 0.4320482365620423),  ('palliative care', 0.40917470170328807),  ('hospice', 0.31786424281906916),  ('life', 0.3074732047948044),  ('end life', 0.29624203180236186),  ('cancer', 0.2949896739323527),  ('eol', 0.28376579338567354),  ('care', 0.2771551591806313),  ('end', 0.24793892545552176),  ('pc', 0.22621811854916965)], | End of Life and Ethics |
| 26 | [('bpd', 0.329515163789722),  ('surfactant', 0.31406683785946565),  ('infants', 0.3111386184351611),  ('pda', 0.2964588979341973),  ('preterm', 0.22053543140435233),  ('weeks', 0.21292046553383392),  ('ncpap', 0.21196653675079258),  ('preterm infants', 0.2081915914526204),  ('bronchopulmonary dysplasia', 0.20760801367349765),  ('bronchopulmonary', 0.20726064565418764)], | Pediatrics Care |
| 27 | [('vap', 0.4377259964073132),  ('ventilator associated', 0.2979313192897275),  ('ventilator', 0.2839692777584477),  ('associated pneumonia', 0.27409203603158006),  ('ventilator associated pneumonia', 0.27281508848837416),  ('pneumonia', 0.2710163858340266),  ('cuff', 0.26922001158888326),  ('bal', 0.2332381597808366),  ('pneumonia vap', 0.22195978289175095),  ('associated pneumonia vap', 0.22040530496931757)], | Ventilation Related |
| 28 | [('mobilization', 0.31253214154152176),  ('mobility', 0.3040090316533657),  ('muscle', 0.2987167340158712),  ('rehabilitation', 0.26565915418480496),  ('early mobilization', 0.24803546833433668),  ('physical', 0.24226964817505608),  ('early', 0.21231299693764347),  ('physiotherapy', 0.20766659028640794),  ('icu', 0.20302647311959443),  ('exercise', 0.20282345598442975)], | Nutrition and Rehabilitation |
| 29 | [('obesity', 0.3184191281531975),  ('covid', 0.2986227736475514),  ('covid 19', 0.29830700274199423),  ('19', 0.2839224095107938),  ('bmi', 0.28168797060033846),  ('obese', 0.2699227794051501),  ('diabetes', 0.2318805748039421),  ('arb', 0.19968818764720014),  ('acei', 0.19692985013819306),  ('obese patients', 0.19455044587594025)], | Obesity |
| 30 | [('peep', 0.31080113712485036),  ('ventilation', 0.2694924868292526),  ('tidal', 0.26471000358786184),  ('pressure', 0.2625114018344906),  ('lung', 0.2517996010996156),  ('tidal volume', 0.23460420168668608),  ('volume', 0.23395526623649027),  ('expiratory', 0.22914595492735654),  ('ards', 0.2192292465512495),  ('ventilator', 0.21779238411659238)]} | Ventilation Related |
