# Supplementary figures and images for: Comparing Open-Access Database and Traditional Intensive Care Studies Using Machine Learning: Bibliometric Analysis Study
Source: J Med Internet Res. 2024 Apr 17;26:e48330. doi: 10.2196/48330 (PMC11063894; doi:10.2196/48330)

**Multimedia Appendix 4:** Similarity matrix of 30 topics.


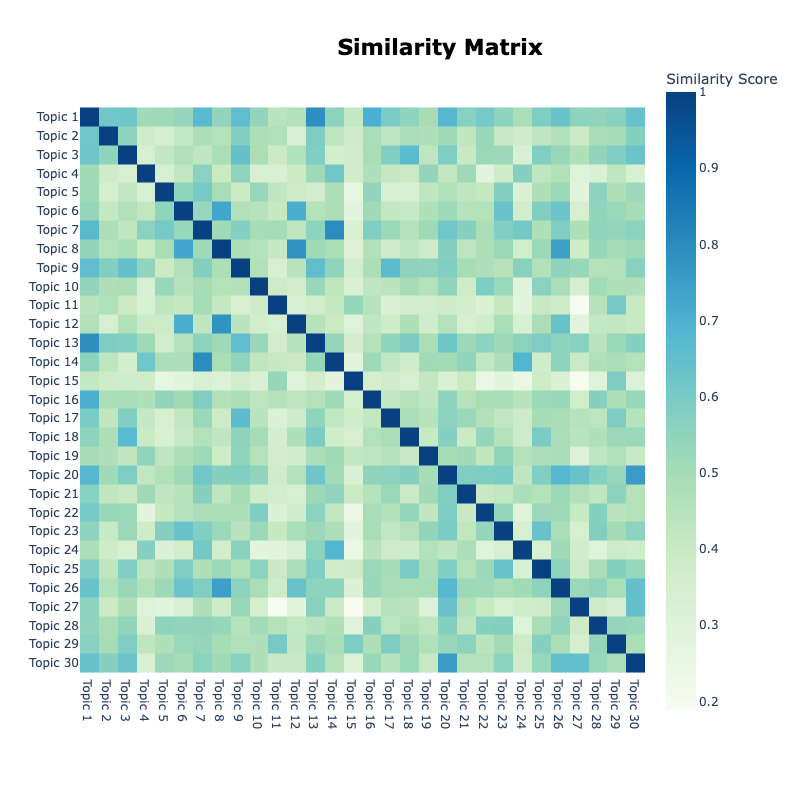

Supplement: Multimedia Appendix 4 [file jmir_v26i1e48330_app4.docx]
